# Supplementary material for: Tracing development of song memory with fMRI in zebra finches after a second tutoring experience
Source: Commun Biol. 2023 Mar 30;6:345. doi: 10.1038/s42003-023-04724-2 (PMC10063632; doi:10.1038/s42003-023-04724-2)
Supplement: Supplementary file 2 — Supplementary Figures [file 42003_2023_4724_MOESM2_ESM.pdf]

Supplementary Material

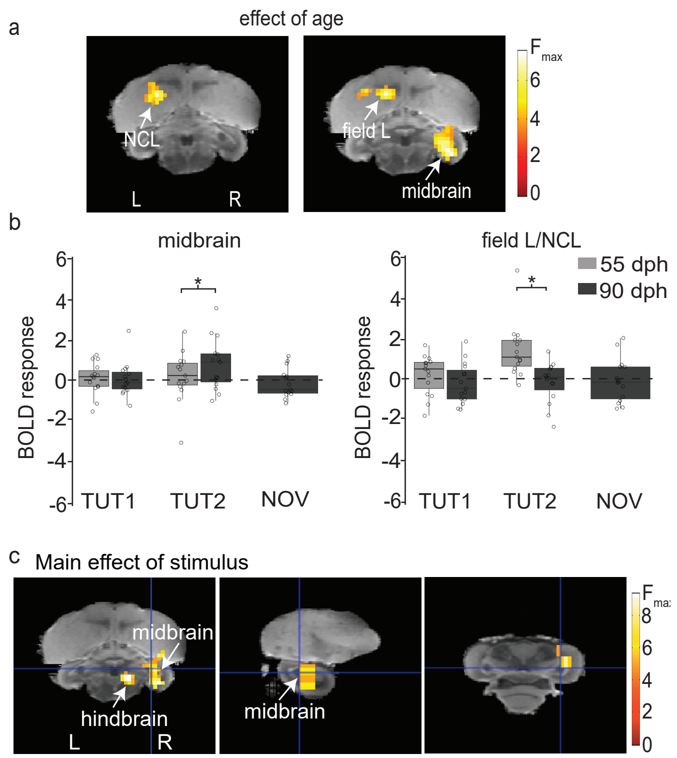

Supplementary Fig 1: BOLD signal changes after experience with the first song tutor (55 dph) and second song tutor (90 dph) in sequentially tutored birds. a) Statistical map of voxels in sequentially tutored birds that are differentially activated in response to song stimuli between 55- and 90-day old birds, showing activated clusters in NCL and field L in the left hemisphere and in the midbrain in the right hemisphere, (F-test from repeated measures ANOVA, N = 15). All voxels with F > 3.16 ($p_{uncorrected} < 0.05$) are displayed. Images represent statistical maps superimposed on coronal sections from the high-resolution zebra finch MRI atlas [1]. b) BOLD response (β weights) for each stimulus relative to rest periods in the clusters indicated with a white arrow in Supplementary Fig 1a. The asterisk indicates statistically significant differences between 55- and 90-day old birds (*$p_{FWE} < 0.05$, N = 15). Boxplots showing the interquartile range (box), median (black line), and 1st and 3rd quartile; individual birds are represented by circles. Abbreviations: (NCL) caudolateral nidopallium; (CMM) caudomedial mesopallium. c) Main effect of stimulus (TUT1, TUT2, and NOV) in 90-day old birds. Statistical map of voxels (F-test from one-way ANOVA within subjects, N = 15, $p_{uncorrected} < 0.01$) overlaid on coronal, sagittal, and axial slices of zebra finch MRI atlas [1]. F-values are color coded according to the scale displayed on the right.

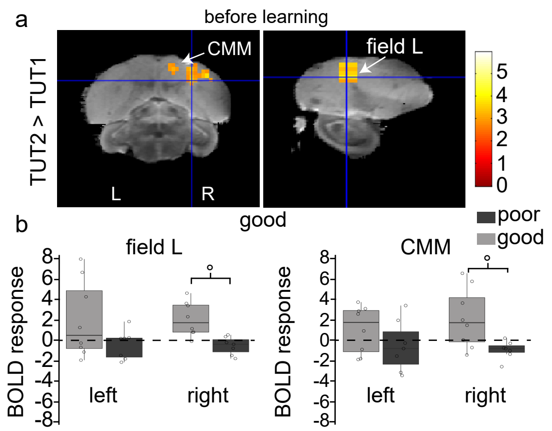

Supplementary Fig 2: a) Before exposure to the second song tutor, there is already a TUT2-selective response (TUT2 > TUT1) in CMM and field L (all voxels with $t > 2.65$, $p_{uncorrected} < 0.01$ are displayed) in juveniles which go on to learn well from the second tutor (good learners). Statistical map of voxels superimposed on a high-resolution MRI atlas of the zebra finch brain [1]. b) Difference in BOLD response (β weights) between different stimuli in the cluster indicated with a white arrow in A before learning the second tutor song. Boxplots showing the interquartile range (box), median (black line), and 1st and 3rd quartile; individual birds are represented by circles (°$p_{uncorrected} < 0.01$, N = 15). Abbreviations: caudomedial mesopallium (CMM)

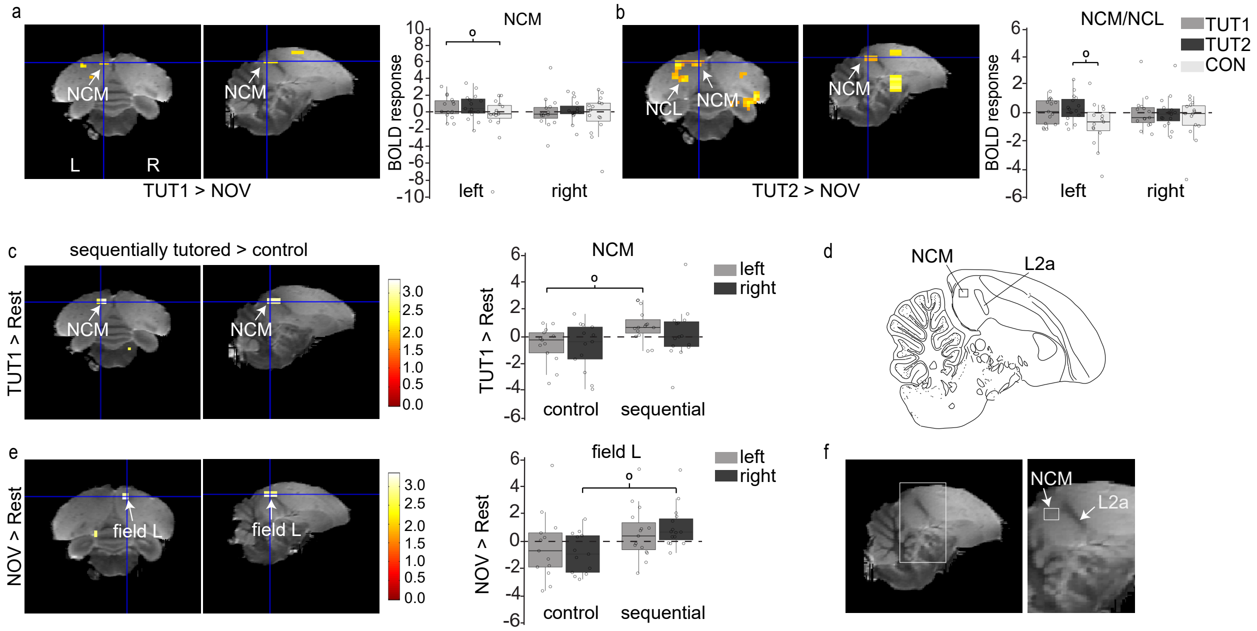

Supplementary Fig 3: a-b) Statistical map of post-hoc t-tests performed within the main effect of stimulus in sequentially tutored birds. All voxels with $t > 1.7$ at $p_{uncorrected} < 0.05$ are displayed. Data is represented as mean ± SEM (°$p_{uncorrected} < 0.05$, N = 15). c) Tutor song elicited a higher BOLD response in the left NCM while conspecific song (e) elicited a higher BOLD response in field L in sequentially tutored birds as compared to control birds. BOLD response (β weights) in NCM and field L clusters indicated with white arrows in Supplementary Fig 3c and 3e and their mirrored counterparts in the other hemisphere. Boxplots

showing the interquartile range (box), median (black line), 1st and 3rd quartile; each circle represents data from one individual bird. d) Line drawing of a parasagittal section from the zebra finch histological atlas [2]. f) Detailed location of the activated cluster in Supplementary Fig 3c. Abbreviations: (MLd) mesencephalicus lateralis dorsalis, (NCM) caudomedial Nidopallium. ($^{o}p_{uncorrected} < 0.01$, control N = 13, sequentially tutored N = 15).

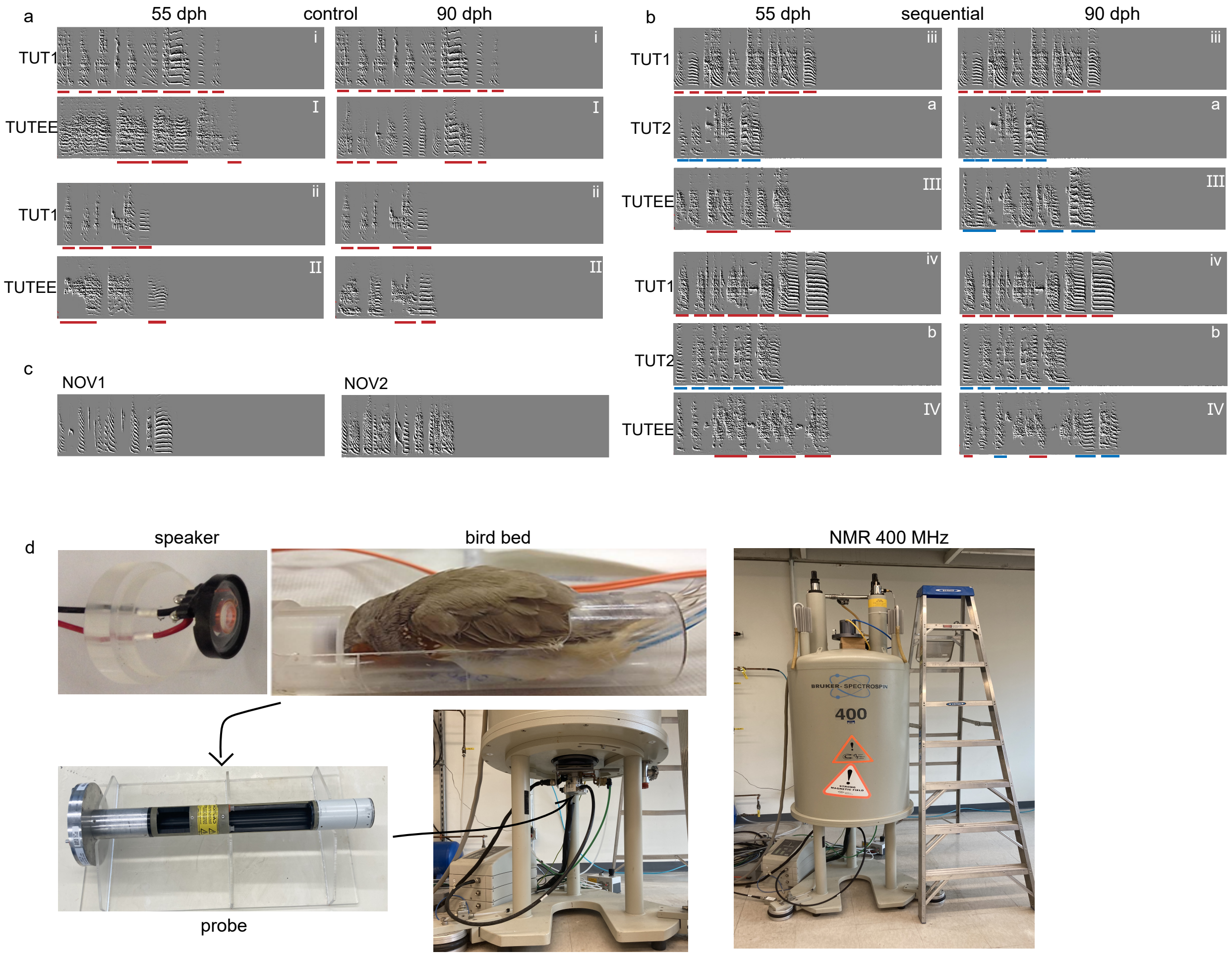

Supplementary Fig 4: a-b) Representative sonograms of control group birds (a) and sequentially tutored birds (b) at 55 and 90 dph along with their first tutor (TUT1) and second tutor (TUT2). c) Representative sonograms of novel conspecific birds (NOV) that were used to compare tutee songs with. Syllables copied from TUT1 and TUT2 are indicated by red and blue respectively. Syllables were scored by human observers on a scale from 0 to 3 (0 being the lowest resemblance to a specific tutor syllable and 3 being highest). Syllables that were scored with either 2 or 3 are indicated by red (TUT1) and blue (TUT2) bars. Uppercase roman numerals represent different tutees, lowercase letters represent the different TUT2 used for

sequential tutoring. Lower case roman numerals represent different TUT1 used for control and sequentially tutored birds. d) Picture of an anesthetized bird restrained in a customized bird bed (holder) with the speaker placed into the holder in front of the bird's head. This bird holder is inserted into the probe, and the probe is inserted with the head facing up from the bottom of the NMR machine.

References

1. Poirier C*, et al.* A three-dimensional MRI atlas of the zebra finch brain in stereotaxic coordinates. *Neuroimage* **41**, 1-6 (2008).
2. Karten HJ*, et al.* Digital atlas of the zebra finch (*Taeniopygia guttata*) brain: A high-resolution photo atlas. *Journal of Comparative Neurology* **521**, 3702-3715 (2013).
