# Supplementary material for: Tracing development of song memory with fMRI in zebra finches after a second tutoring experience
Source: Commun Biol. 2023 Mar 30;6:345. doi: 10.1038/s42003-023-04724-2 (PMC10063632; doi:10.1038/s42003-023-04724-2)
Supplement: Supplementary file 5 — Reporting Summary [file 42003_2023_4724_MOESM5_ESM.pdf]

nature portfolio

Corresponding author(s): Gobes, SMH

Last updated by author(s): Feb 27, 2023

Reporting Summary

Nature Portfolio wishes to improve the reproducibility of the work that we publish. This form provides structure for consistency and transparency in reporting. For further information on Nature Portfolio policies, see our Editorial Policies and the Editorial Policy Checklist.

Statistics

For all statistical analyses, confirm that the following items are present in the figure legend, table legend, main text, or Methods section.

n/a	Confirmed	
☐	☒	The exact sample size (n) for each experimental group/condition, given as a discrete number and unit of measurement
☐	☒	A statement on whether measurements were taken from distinct samples or whether the same sample was measured repeatedly
☐	☒	The statistical test(s) used AND whether they are one- or two-sided<br>*Only common tests should be described solely by name; describe more complex techniques in the Methods section.*
☒	☐	A description of all covariates tested
☐	☒	A description of any assumptions or corrections, such as tests of normality and adjustment for multiple comparisons
☐	☒	A full description of the statistical parameters including central tendency (e.g. means) or other basic estimates (e.g. regression coefficient) AND variation (e.g. standard deviation) or associated estimates of uncertainty (e.g. confidence intervals)
☐	☒	For null hypothesis testing, the test statistic (e.g. F, t, r) with confidence intervals, effect sizes, degrees of freedom and P value noted<br>*Give P values as exact values whenever suitable.*
☒	☐	For Bayesian analysis, information on the choice of priors and Markov chain Monte Carlo settings
☒	☐	For hierarchical and complex designs, identification of the appropriate level for tests and full reporting of outcomes
☒	☐	Estimates of effect sizes (e.g. Cohen's d, Pearson's r), indicating how they were calculated

Our web collection on statistics for biologists contains articles on many of the points above.

Software and code

Policy information about availability of computer code

Data collection	Paravision 4.0 for data collection
Data analysis	SPM 12, ANTS, FSL, R version 3.5.1

For manuscripts utilizing custom algorithms or software that are central to the research but not yet described in published literature, software must be made available to editors and reviewers. We strongly encourage code deposition in a community repository (e.g. GitHub). See the Nature Portfolio guidelines for submitting code & software for further information.

Data

Policy information about availability of data

All manuscripts must include a data availability statement. This statement should provide the following information, where applicable:

- Accession codes, unique identifiers, or web links for publicly available datasets
- A description of any restrictions on data availability
- For clinical datasets or third party data, please ensure that the statement adheres to our policy

The datasets generated during the current study are available from the corresponding author upon request

Field-specific reporting

Please select the one below that is the best fit for your research. If you are not sure, read the appropriate sections before making your selection.

☒ Life sciences ☐ Behavioural & social sciences ☐ Ecological, evolutionary & environmental sciences

For a reference copy of the document with all sections, see nature.com/documents/nr-reporting-summary-flat.pdf

Life sciences study design

All studies must disclose on these points even when the disclosure is negative.

Sample size	No sample size calculation was performed; based on other fMRI studies in birds we had a similar success rate in obtaining a BOLD response in our subjects.
Data exclusions	Four subjects were excluded: one subject had head motion > 0.5 mm, two subjects did not show any BOLD activation, one subject showed BOLD activation in only one of the sessions and was excluded from ANOVA as well as from that individual session. The exclusion criteria was predetermined based on the literature.
Replication	28 out of 32 subjects consistently showed activation in auditory regions.
Randomization	Animals were allocated randomly to each group.
Blinding	Blinding was not possible because the experiment was longitudinal and the same animals had to be imaged again during their development, which was done by the same person to keep the data acquisition process the same for all animals.

Reporting for specific materials, systems and methods

We require information from authors about some types of materials, experimental systems and methods used in many studies. Here, indicate whether each material, system or method listed is relevant to your study. If you are not sure if a list item applies to your research, read the appropriate section before selecting a response.

Materials & experimental systems

n/a	Involved in the study	
☒	☐	Antibodies
☒	☐	Eukaryotic cell lines
☒	☐	Palaeontology and archaeology
☐	☒	Animals and other organisms
☒	☐	Human research participants
☒	☐	Clinical data
☒	☐	Dual use research of concern

Methods

n/a	Involved in the study	
☒	☐	ChIP-seq
☒	☐	Flow cytometry
☐	☒	MRI-based neuroimaging

Animals and other organisms

Policy information about studies involving animals; ARRIVE guidelines recommended for reporting animal research

Laboratory animals	Zebra finch (Taeniopygia guttata), male, juveniles (55 day old), and adults (90 day old).
Wild animals	n/a
Field-collected samples	n/a
Ethics oversight	IACUC protocol #1405, #1702, #2004

Note that full information on the approval of the study protocol must also be provided in the manuscript.

Magnetic resonance imaging

Experimental design

Design type	fMRI block design
Design specifications	55 day old sessions: ON-OFF block design, 32 seconds for ON periods, 32 seconds for OFF periods, 50 ON (25 per stimulus type), 50 OFF blocks

	90-day old sessions: ON-OFF blocks, 32 seconds for ON periods, 32seconds for OFF periods, 75 ON 25 per stimulus type, 75 OFF blocks. Two additional dummy scans were added after every OFF block but were not used for analysis.
Behavioral performance measures	No behavioral measures were recorded during fMRI sessions.

Acquisition

Imaging type(s)	functional
Field strength	9.4T
Sequence & imaging parameters	spin echo, for functionals scans: fov:25 x25x11.25mm3, matrix: 64x64, interslice gap: 0.75mm, spatial resolution: 0.39 x 0.39 mm2, orientation: axial, TEeff/TR: 60/2000 ms<br>For anatomical scans: fov: 25x25x11.25mm3, matrix: 256x256, interslice gap: 0.75mm, resolution: 0.097x0.097mm2, orientation: axial, TEeff/TR: 60/3000 ms
Area of acquisition	whole brain

Diffusion MRI ☐ Used ☒ Not used

Preprocessing

Preprocessing software	SPM 12 for preprocessing, smoothing kernel size: 0.5mm FWHM was used. The SPM smoothing function was used to smooth functional images.
Normalization	Functional scans were realigned using six-parameter rigid body spatial transformation in SPM12. The realigned fMRI scans were then co-registered to their own 3-D RARE anatomical scan using affine registration with the FLIRT tool in the data processing software "FMRIB Software Library" (FSL, https://fsl.fmrib.ox.ac.uk/fsl/). In parallel, the 3-D RARE anatomical scan of each subject was spatially normalized with the high resolution zebra finch MRI atlas using ANTS. function used "SyN": Symmetric normalization: Affine + deformable transformation, with mutual information as optimization metric (http://stnava.github.io/ANTs/). The transformation matrix generated in the previous step was then applied to the realigned and co-registered functional data using ANTs(http://stnava.github.io/ANTs/) using linear affine transformation.
Normalization template	Data was normalized to zebra finch MRI atlas space.
Noise and artifact removal	Any subject with a head motion > 0.5mm was removed from the study. Six estimated movement parameters derived from the realignment corrections included as regressors in the model to account for residual head movement. A high pass filter of 320 seconds was applied to remove low frequency drifts in the BOLD signal.
Volume censoring	not applied

Statistical modeling & inference

Model type and settings	Statistical voxel-based analyses were performed using a mass-univariate approach based on the general linear model implemented in SPM12. First level: a general linear model convolved with the canonical hemodynamic response function was applied.<br>At the second level: group level random effect analysis was done.
Effect(s) tested	Repeated measures ANOVA (flexible factorial design in SPM12) with subjects as random factor was used to determine the interaction between the age of the birds (55 and 90 dph) and the stimulus presented (TUT1, TUT2, NOV).<br>The main effect of stimulus in 90-day old birds was measured using One-way-ANOVA within subjects and subsequent one-tailed t-tests were done to determine differences between stimuli (TUT2 > TUT1, TUT2 > NOV, TUT1 > TUT2, TUT1 > NOV, NOV > TUT1, NOV > TUT2).<br>The main effect of stimulus in 55-day old birds was determined by using paired-t-test. One-tailed t-tests were done to find differences between stimuli (TUT > NOV, NOV > TUT)

Specify type of analysis: ☐ Whole brain ☐ ROI-based ☒ Both

Anatomical location(s)	Anatomical locations for specific brain nuclei were determined using the zebra finch MRI atlas
Statistic type for inference (See Eklund et al. 2016)	voxel-wise
Correction	FWE

Models & analysis

n/a	Involved in the study
☒	☐ Functional and/or effective connectivity
☒	☐ Graph analysis
☒	☐ Multivariate modeling or predictive analysis
